# Global Spread of Multiple Aminoglycoside Resistance Genes

**DOI:** 10.3201/eid1106.040924

**Published:** 2005-06

**Authors:** Kunikazu Yamane, Jun-ichi Wachino, Yohei Doi, Hiroshi Kurokawa, Yoshichika Arakawa

**Affiliations:** * National Institute of Infectious Diseases, Tokyo, Japan

**Keywords:** plasmid, resistance, transposon, 16S rRNA methylase, aminoglycoside

## Abstract

Emergence of the newly identified 16S rRNA methylases RmtA, RmtB, and ArmA in pathogenic gram-negative bacilli has been a growing concern. ArmA, which had been identified exclusively in Europe, was also found in several gram-negative pathogenic bacilli isolated in Japan, suggesting global dissemination of hazardous multiple aminoglycoside resistance genes.

Multidrug-resistant gram-negative super microbes have been emerging worldwide. Since carbapenems and fluoroquinolones are the last resort against infections caused by gram-negative bacilli (1,2), the proliferation and dissemination of such clinical isolates that produce metallo-β-lactamases and acquire mutations in *gyrA* and *parC* genes have become a global threat (3,4). Aminoglycosides, including amikacin and tobramycin, are still potent agents for use against resistant bacilli. One of the most common resistance mechanisms against aminoglycosides is the production of aminoglycoside-modifying enzymes, such as aminoglycoside acetyltransferases, aminoglycoside phosphorylases, and aminoglycoside adenyltransferases (5), which are mainly mediated by transferable large plasmids.

Recently, a series of special methylases that protect microbial 16S rRNA, the main target of aminoglycosides, was identified in several nosocomial pathogens, including *Pseudomonas aeruginosa* (6), *Serratia marcescens* (7), and *Klebsiella pneumoniae* (8). The newly identified 16S rRNA methylases RmtA and RmtB were reported from Japan in 2003 and 2004, respectively (6,7). The gene for ArmA was initially sequenced in *Citrobacter freundii* isolated in Poland (GenBank accession no. AF550415) and later characterized in *K. pneumoniae* isolated in France in 2003 (8). In 2004, nosocomial spread of ArmA- or RmtB-producing *Escherichia coli* and *K. pneumoniae* was reported from Taiwan (9).

These enzymes are capable of conferring an extraordinary high level of resistance (MIC >512 mg/L) against most clinically important aminoglycosides as was observed among aminoglycoside-producing actinomycetes, suggesting their probable phylogenic relationship with the intrinsic 16S rRNA methylases of actinomycetes (Figure). RmtA shared 82% amino acid identity with RmtB, but the amino acid sequence similarities between 16S rRNA methylases isolated from pathogenic gram-negative microbes and those from aminoglycoside-producing actinomycetes were relatively low (≤33%). From analyses of the genetic environments of genes encoding 16S rRNA methylases, the *rmtA* gene is likely associated with the mercury-resistant transposon Tn*5041* (10); the *rmtB* gene was found in the flanking region of Tn*3*-like structure (7). The *armA* gene was found on a large plasmid which carries a type 1 integron (8) that mediates various gene cassettes responsible for multiple antimicrobial resistance. The structure of these genetic environments implied that the genes for these 16S rRNA methylases are mediated by mobile genetic elements carried by transferable large plasmids (7,8,10). In fact, the *rmtA* gene was transferred from *P. aeruginosa* strain AR-2 to an aminoglycoside-susceptible *P. aeruginosa* strain 105 by conjugation in vitro (6). The *rmtB* gene was also transferred from *S. marcescens* S-95 to *E. coli* by transformation (7). The *armA* gene was located on a composite transposon Tn*1548* (11).

**Figure:**
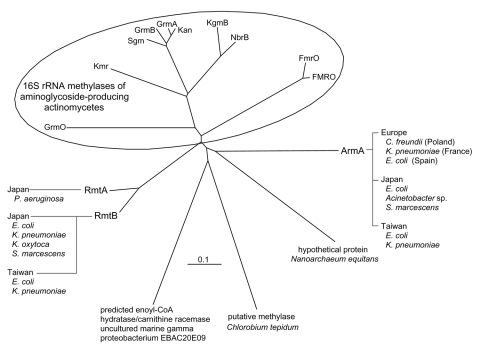
Phylogenic relationship among the 16S rRNA methylases. Each amino acid sequence was subjected to the analysis referred to the following sources: FmrO, accession no. JN0651; Kmr, accession no. AB164642; GrmA, accession no. M55520; GrmB, accession no. M55521; GrmO, accession no. AY524043; Kan, accession no. AJ414669; Sgm, accession no. A45282; KgmB, accession no. S60108; NbrB, accession no. AF038408; FMRO, Q08325; RmtA, (6); RmtB, (7); ArmA, (8); predicted enoyl-CoA hydratase/carnithine racemase of uncultured marine gamma proteobacterium EBAC20E09, accession no. AAS73112; putative methylase of *Chlorobium tepidum*, accession no. AAM72273; hypothetical protein of *Nanoarchaeum equitans*, accession no. AAR39385. The ClustalW program provided by the DNA Data Bank of Japan (http://www.ddbj.nig.ac.jp/search/clustalw-e.html) was used in this study.

Thus, the growing concern was that these newly identified aminoglycoside-resistance genes could easily spread and be further disseminated among the glucose-nonfermentative gram-negative bacilli, including *P. aeruginosa* and *Acinetobacter* spp. and the genera belonging to the family *Enterobacteriaceae*.

## The Study

We conducted a preliminary screening of the 16S rRNA methylase-producing bacilli on our gram-negative microbial stock of 2,877 strains isolated from Japanese hospitals within the past several years. Arbekacin, a semisynthetic aminoglycoside belonging to the kanamycin group, requires 2 modifications at the (6´) aminogroup and the (2´´) hydroxyl group for inactivation, so this agent is not inactivated by known plasmid-mediated aminoglycoside-modifying enzymes. Therefore, a high-level arbekacin resistance (MIC >512 mg/L) was used as a marker for screening the 16S rRNA methylase-producing strains. All arbekacin-resistant strains were subjected to polymerase chain reaction (PCR) analysis to detect *rmtA*, *rmtB*, or *armA*, and all strains were PCR positive, except for a strain of *Acinetobacter* demonstrating a very high level of resistance to arbekacin (MIC 1,024 mg/L). This strain was later shown to produce both aminoglycoside 6´-acetyltransferase and 2´´-adenyltransferase (12), so arbekacin was inactivated in this strain by both 6´-acetylation and 2´´-adenylation. Each PCR primer set was used to detect *rmtA* and *rmtB* genes as in our previous reports (6,7). The PCR primers for amplification of *armA* were newly designed (forward: 5´-AGG TTG TTT CCA TTT CTG AG-3´, reverse: 5´-TCT CTT CCA TTC CCT TCT CC-3´), and the predicted size of the amplicon was 590 bp. These 3 sets of PCR primers were very reliable in detecting *rmtA*, *rmtB*, and *armA* genes, respectively. Each PCR amplicon was then subjected to sequencing analyses on both strands to confirm its nucleotide sequences for detecting mutations in the methylase genes.

As reported in our previous study, *rmtA* and *rmtB* genes had been found in *P. aeruginosa* isolates (6,10) and in 1 strain of *S. marcescens* (7), respectively. As shown in the Table, 5 *P. aeruginosa* strains isolated after our previous report (6) were *rmtA* positive. The *rmtB* gene was additionally identified in 4 *K. pneumoniae*, 2 *E. coli*, and 1 *K. oxytoca* strains in Japan. To our surprise, the *armA* gene, which had been found in various gram-negative microbial species belonging to the family *Enterobacteriaceae* exclusively in Europe as reported by Galimand et al. (13), was also identified in Japan in 1 strain each of *E. coli*, *S. marcescens*, and *Acinetobacter* sp. Notably, the *armA* and *rmtB* genes were also recently identified in *K. pneumoniae* and *E. coli* in Taiwan (9). Furthermore, the genetic environment of the *armA* gene found in *C. freundii* isolated in Poland was similar to that of *K. pneumoniae* isolated in France. The genetic environments of the *armA* gene found in the 3 Japanese microbial species, *E. coli*, *S. marcescens*, and *Acinetobacter* sp., (GenBank accession nos. AB116388 and AB117519), were also similar to those found in Europe (GenBank accession nos. AF550415 and AY220558). These findings suggest that the ArmA-producing gram-negative nosocomial microbes that harbor a very similar genetic environment carrying the *armA* gene have spread globally.

**Table:** Methylase-producing strains of 16S rRNA identified after previous study (6)

Species and strain	Type	Year of isolation	Hospital	Prefecture
*Pseudomonas aeruginosa* P122	RmtA	2002	A	Aichi
*P. aeruginosa* P340	RmtA	2002	B	Gifu
*P. aeruginosa* 02-386	RmtA	2002	C	Saitama
*P. aeruginosa* 03-29	RmtA	2003	D	Aichi
*P. aeruginosa* 03-230	RmtA	2003	E	Shizuoka
*Escherichia coli* 01-139	RmtB	2001	H	Yamanashi
*Klebsiella pneumoniae* 01-140	RmtB	2001	H	Yamanashi
*Klebsiella oxytoca* 01-141	RmtB	2001	H	Yamanashi
*K. pneumoniae* 01-142	RmtB	2001	H	Yamanashi
*E. coli* C316	RmtB	2002	F	Hyogo
*Serratia marcescens* S95	RmtB	2002	G	Kohchi
*K. pneumoniae* 03-252	RmtB	2003	H	Yamanashi
*K. pneumoniae* 03-518	RmtB	2003	H	Yamanashi
*E. coli* C316-2	ArmA	2003	F	Hyogo
*S. marcescens* ARS8	ArmA	2003	I	Tochigi
*Acinetobacter* sp. ARS6	ArmA	2003	J	Kanagawa


## Conclusions

As described previously, arbekacin still shows a very broad antimicrobial spectrum from gram-positive to gram-negative nosocomial microbes and has been approved solely to treat methicillin-resistant *Staphylococcus aureus* (MRSA) infections in Japan since 1990 to ensure the prudent use of this agent. The emergence and presence of the 16S rRNA methylase-producing gram-negative bacilli, however, has not been well recognized in Japan to date; arbekacin has not been listed among the antimicrobial agents for daily antimicrobial susceptibility testing of gram-negative microbes.

The use of semisynthetic aminoglycosides, including arbekacin, in Japanese clinical settings for >10 years may have promoted the emergence and dissemination of the 16S rRNA methylase-producing gram-negative microbes in Japan. The large amount of various aminoglycosides used in livestock-farming environments could have also been a selective pressure for the emergence and spread of pathogenic microbes that harbor genetic determinants for the newly identified 16S rRNA methylases, as exemplified by recent isolation of ArmA-producing *E. coli* from swine in Spain (GenBank accession no. AY522431).

Since acquisition of multidrug resistance against clinically important antimicrobial agents such as carbapenems and fluoroquinolones has been developing rapidly worldwide, the acceleration of even greater aminoglycoside resistance among gram-negative bacilli promises to become an actual clinical concern in the near future, just as vancomycin-resistant enterococci (VRE) did in the 1990s (14). The emergence of gram-positive cocci including MRSA and VRE that acquire the 16S rRNA methylase could also be a grave clinical matter, although fortunately no such hazardous microbes have been identified. Thus, steps must be taken to further block proliferation of these multidrug-resistant gram-negative super microbes, including *P. aeruginosa*, *K. pneumoniae*, and *Acinetobacter* spp., as well as multidrug-resistant cocci such as MRSA and VRE, which have acquired an extraordinarily high level of resistance to various aminoglycosides through production of 16S rRNA methylases, especially in clinical environments.
